# Impact of changing donor human milk feeding guideline for extremely preterm infants on the use of infant formula and cost of donor human milk purchase

**DOI:** 10.1038/s41372-024-02182-0

**Published:** 2024-11-20

**Authors:** Yingying Zheng, Jennifer Fowler, Chance Rector, Dmitry Tumin, Maja Herco

**Affiliations:** 1 Department of Neonatology, East Carolina University Health Medical Center, Greenville, NC USA; 2 Department of Clinical Nutrition Services, East Carolina University Health Medical Center, Greenville, NC USA; 3 Brody School of Medicine, East Carolina University, Greenville, NC USA; 4 Department of Pediatrics, Brody School of Medicine, East Carolina University, Greenville, NC USA

**Keywords:** Paediatrics, Outcomes research

## Introduction

Donor human milk has become an essential part of feeding premature infants due to its association with a lower incidence of necrotizing enterocolitis (NEC) and late-onset sepsis [1, 2]. Many institutions have implemented policies to expand the use of donor human milk, but few studies have analyzed the impact on patients’ feeding after such policy changes. We analyzed how implementing a new donor human milk guideline at our institution influenced donor human milk use among extremely preterm infants.

## Methods

The East Carolina University Health Medical Center Neonatal Intensive Care Unit (NICU) changed the donor human milk guideline in 2022. Previously, all infants with birthweight <1500 g received 30 days of donor human milk regardless of gestational age. After September 15th, 2022, all neonates born <32 weeks and <1800 grams qualified to receive donor human milk until 32 weeks corrected gestational age (GA). Following Institutional Review Board approval, we retrospectively identified infants born between 22 and 28 weeks of GA admitted to our NICU between September 2021 and September 2023, who were then divided into a pre-intervention epoch (September 2021–August 2022) and a post-intervention epoch (October 2022-September 2023). The less than 28 weeks GA (<28GA) group consisted of infants between 22 weeks 0 days and 27 weeks 6 days of GA, as this group of patients would have been eligible to receive a few additional weeks’ supply of donor human milk following the intervention. Infants born between 28 weeks 0 day to 28 weeks 6 days of GA were in the 28 weeks GA (28GA) group, as this group of patients received the same duration of donor human milk regardless of the guideline. The primary outcome was the number of days on donor human milk until 32 weeks of corrected gestational age (CA) was reached. Secondary outcomes included weight, length, and head circumference at 32 weeks CA. We also obtained data on the cost of donor human milk to the institution over the study period. Data were summarized as medians with interquartile ranges (IQR) or counts with percentages. We compared study variables between periods in each gestational age group using rank-sum tests, Chi-square tests, or Fisher’s exact tests, as applicable. Data analyzes were performed using Stata/SE 16.1 (College Station, TX: StataCorp, LP), and P < 0.05 was considered statistically significant.

## Results

The pre-intervention <28GA group included 34 patients, the post-intervention <28GA group included 44 patients, the pre-intervention 28GA group included 16 patients, and the post-intervention 28GA group included 19 patients. There was no difference in maternal characteristics, neonatal characteristics, and feeding parameters between the groups (Appendix I and II). In the <28GA group, the median of total days on donor human milk increased from 27 (IQR 9–29) to 28.5 (IQR 21.8–36.5) after guideline implementation, but this change was not statistically significant (p = 0.3). Meanwhile, the use of the formula in the <28GA group decreased significantly after the protocol change (Table 1). More patients received maternal breast milk in the post-intervention <28GA group. In addition, the total cost of donor human milk for all infants decreased from approximately $83000 to $54000 after the protocol change. There was no difference in the weight, length, or head circumference z-scores at 32 weeks corrected gestational age associated with the guideline change in either cohort (Table 1).

**Table 1:** Feeding Types and Z-scores of the infants.

Variable	Pre-intervention < 28GA group Group (n = 34)	Post-intervention < 28GA group group (n = 44)	P-value Intervention < 28GA group	Pre-intervention 28GA group (n = 16)	Post-intervention 28GA group (n = 19)	P-value 28GA group
	N (%) or median (IQR)	N (%) or median (IQR)		N (%) or median (IQR)	N (%) or median (IQR)	
Feeding type (admission-32 WKS CA^a^)						
			0			0.08
DHM only	0 (0%)	12 (27%)		3 (19%)	7 (37%)	
MOM^b^, DHM^c^ and formula	20 (59%)	1 (2%)		1 (6%)	1 (5%)	
DHM^c^ and formula	11 (32%)	0 (0%)		0 (0%)	0 (0%)	
MOM^b^ and formula	1 (3%)	0 (0%)		0 (0%)	0 (0%)	
MOM^b^ and DHM^c^	2 (6%)	31 (70%)		12 (75%)	11 (58%)	
Z-scores						
Birth weight z-score	0.075 (−0.19, 0.62)	0.41 (−0.085, 0.85)	0.628	0.21 (−0.14, 0.77)	0.25 (−0.28, 0.81)	0.896
32 WKS CA^a^ weight z-score	−0.725 (−0.94, −0.49)	−0.89 (−1.26, −0.38)	0.2	−0.285 (−0.6, 0.035)	−0.59 (−0.94, −0.16)	0.226
Birth length z-score	0.065 (−0.28, 0.77)^d^	0.22 (−0.53, 0.8)^e^	0.872	0.885 (−0.40, 1)	0.23 (−0.34, 0.88)	0.477
32 WKS ± 6D CA^f^ length z-score	−1.34 (−1.7, −0.81)^g^	−1.255 (−2.1, −0.895)^h^	0.515	−0.21 (−0.79, 0)^i^	−0.785 (−1.5, -0.23)^j^	0.060
Birth head circumference z-score	−0.31 (−0.55, −0.31)^k^	−0.22 (−0.56, 0.39)^l^	0.88	0.235 (−0.32, 1.05)	−0.28 (−0.45, 0.375)	0.254
32 WKS ± 6D head CA^f^ circumference z-socre	−1.35 (−2.01, −0.73)^m^	−1.215 (−2.28, −0.635)^n^	0.841	−0.925 (−1.63, −0.6)^o^	−1.03 (−1.4, −0.05)^p^	0.727

^a^32 weeks 0 day corrected age.

^b^*MOM* maternal breast milk.

^c^*DHM* Donor human milk.

^d^Missing data of 1 patient.

^e^Missing data of 3 patient.

^f^32 weeks ±6 days of corrected age.

^g^Missing data of 10 patients.

^h^Missing data of 8 patients.

^i^Missing data of 3 patients.

^j^Missing data of 1 patient.

^k^Missing data of 2 patients.

^l^Missing data of 3 patients.

^m^Missing data of 9 patients.

^n^Missing data of 6 patients.

^o^Missing data of 2 patients.

^p^Missing data of 1 patient.

## Discussion

Implementing a new feeding guideline that allowed for longer donor human milk use in the extremely preterm population did not significantly increase donor human milk use but dramatically decreased formula use, a pattern described in other studies on extending donor human milk utilization [3]. In this study, we collected data on the cost of donor human milk for all infants, not limited to the infants included in the study. We also found that the total cost of donor human milk decreased after the protocol change, likely due to the weight-based nature of breast milk use and shorter utilization of donor human milk in patients born at >29 weeks of gestational age. Since the incidence of NEC is higher in extremely premature infants than in other infants [4, 5], this group would benefit most from donor human milk, and using gestational age-based criteria for donor human milk eligibility can support appropriate donor human milk access among extremely premature infants while controlling the total cost of donor human milk. Some limitations of this study include the small sample size and the inability to determine causation between the variables and outcomes. Multicenter studies may be considered to further analyze the impact of feeding guideline or protocol changes in extremely premature infants.

## Supplementary information

Appendix I

Appendix II
