# Supplementary material for: Impact of changing donor human milk feeding guideline for extremely preterm infants on the use of infant formula and cost of donor human milk purchase
Source: J Perinatol. 2024 Nov 20;45(5):665–7. doi: 10.1038/s41372-024-02182-0 (PMC12221962; doi:10.1038/s41372-024-02182-0)
Supplement: Supplementary file 1 — Appendix I [file 41372_2024_2182_MOESM1_ESM.docx]

**Supplementary information:**

Appendix I: We summarized the neonatal and maternal characteristics in Appendix I. Appendix I will be submitted as a Microsoft Word document

**Appendix I.** **Maternal and Neonatal Characteristics (N=102)**

| Variable | **Pre-intervention**  **< 28GA group**  **(n=34)** | **Post-intervention**  **< 28GA group**  **(n=44)** | **P value**  **Intervention**  **< 28GA group** | **Pre-intervention 28GA group (n=16)** | **Post-intervention 28GA group (n=19)** | **P value**  **28GA group** |
| --- | --- | --- | --- | --- | --- | --- |
|  | N (%) or median (IQR) | N (%) or median (IQR) |  | N (%) or median (IQR) | N (%) or median (IQR) |  |
| ***Maternal*** | | | | | | |
| Age (years) | 27 (23, 34) | 28 (24, 32) | 0.69 | 29 (28, 34) | 30 (27, 35) | 0.88 |
| Chorioamnionitis | 5 (15%) | 8 (18%) | 0.68 | 2 (12.5%) | 0 (0%) | 0.202 |
| Pre-eclampsia | 4 (12%) | 4 (9%) | 0.723 | 2 (12.5%) | 8 (42%) | 0.071 |
| ***Neonatal*** | | | | | | |
| Sex |  |  | 0.170 |  |  | 0.318 |
| Male | 14 (41%) | 26 (59%) |  | 7 (44%) | 12 (63%) |  |
| Female | 20 (59%) | 18 (41%) |  | 9 (56%) | 7 (37%) |  |
| Birthweight (g) | 859 (700, 975) | 895 (708, 991) | 0.535 | 1165 (1064, 1388) | 1140 (1055, 1250) | 0.582 |
| Apgar at 1 minutes of life | 4 (3, 6) | 3 (2, 5) | 0.208 | 5 (4, 6) | 5 (2, 6) | 0.757 |
| Apgar at 5 minutes of life | 7 (6, 8) | 6 (5, 7) | 0.444 | 7 (6, 8) | 7 (6, 8) | 0.478 |
| IUGR | 4 (12%) | 4 (9%) | 0.723 | 1 (6%) | 3 (16%) | 0.608 |
| Multiple birth | 3 (9%) | 8 (18%) | 0.331 | 5 (31%) | 3 (16%) | 0.424 |
| Race |  |  | 0.404 |  |  | 0.939 |
| Black | 20 (59%) | 25 (57%) |  | 10 (63%) | 11 (58%) |  |
| White | 9 (26%) | 10 (23%) |  | 3 (19%) | 5 (26%) |  |
| Hispanic | 4 (12%) | 3 (7%) |  | 1 (6%) | 1 (5%) |  |
| Other | 1 (3%) | 6 (13%) |  | 2 (12%) | 2 (11%) |  |
| Gestational age at birth |  |  |  |  |  |  |
| 22w0d-22w6d | 1 (3%) | 1 (2%) |  | 0 (0%) | 0 (0%) |  |
| 23w0d-23w6d | 2 (6%) | 2 (5%) |  | 0 (0%) | 0 (0%) |  |
| 24w0d-24w6d | 5 (15%) | 8 (18%) |  | 0 (0%) | 0 (0%) |  |
| 25w0d-25w6d | 5 (15%) | 11 (25%) |  | 0 (0%) | 0 (0%) |  |
| 26w0d-26w6d | 9 (26%) | 11 (25%) |  | 0 (0%) | 0 (0%) |  |
| 27w0d-27w6d | 12 (35%) | 11 (25%) |  | 0 (0%) | 0 (0%) |  |
| 28w0d-28w6d | 0 (0%) | 0 (0%) |  | 16 (100%) | 19 (100%) |  |
| Gestational age at discharge (days) | 40.8 (39, 44) | 41.2 (39, 44) | 0.711 | 38.7 (37, 41) | 38.4 (37, 39) | 0.347 |
| Duration of hospitalization | 97.5 (84, 129) | 102.5 (64, 85) | 0.549 | 73 (91, 131) | 69 (61, 74) | 0.327 |
