# Supplementary material for: Impact of changing donor human milk feeding guideline for extremely preterm infants on the use of infant formula and cost of donor human milk purchase
Source: J Perinatol. 2024 Nov 20;45(5):665–7. doi: 10.1038/s41372-024-02182-0 (PMC12221962; doi:10.1038/s41372-024-02182-0)
Supplement: Supplementary file 2 — Appendix II [file 41372_2024_2182_MOESM2_ESM.docx]

**Supplementary information:**

Appendix II: The feeding parameters are summarized in Appendix II. Appendix II will be submitted as a Microsoft Word document

**Appendix II. Feeding Parameters (N=102)**

| Variable | **Pre-intervention**  **< 28GA group**  **(n=34)** | **Post-intervention**  **< 28GA group**  **(n=44)** | **P value**  **Intervention**  **< 28GA group** | **Pre-intervention 28GA group (n=16)** | **Post-intervention 28GA group (n=19)** | **P value 28GA group** |
| --- | --- | --- | --- | --- | --- | --- |
|  | N (%) or median (IQR) | N (%) or median (IQR) |  | N (%) or median (IQR) | N (%) or median (IQR) |  |
| Feeding start time (days) | 2 (1-2) | 2 (2-2) | 0.322 | 2 (2-2) | 2 (2-2) | 0.756 |
| Time to reach the full feed (days) | 11 (11-13) | 12 (11-14) | 0.477 | 12 (11-13) | 11 (11-13) | 0.509 |
| Cumulative days of NPO^a^ | 2 (1-3) | 1.5 (1-3) | 0.865 | 1 (1-1) | 1 (1-1) | 0.96 |
| The number of patients on >= 25 kcal/oz BM* | 14 (41%) | 14 (32%) | 0.477 | 0 (0%) | 4 (21%) | 0.109 |
| The number of patients whose feeding volume were over 160 ml/kg/day | 3 (9%) | 2 (5%) | 0.648 | 1 (6%) | 0 (0%) | 0.457 |

^a^ NPO: nothing by mouth

* max calories that patient ever received, not the average
